# Beta-Adrenergic Antagonist Tolerance in Amyloid Cardiomyopathy

**DOI:** 10.3389/fcvm.2022.907597

**Published:** 2022-07-11

**Authors:** Stuart Ramsell, Carlos Arias Bermudez, Cyril Ayuk Mbeng Takem Baiyee, Brandon Rodgers, Samir Parikh, Salem Almaani, Nidhi Sharma, Samantha LoRusso, Miriam Freimer, Elyse Redder, Naresh Bumma, Ajay Vallkati, Yvonne Efebera, Rami Kahwash, Courtney M. Campbell

**Affiliations:** ^1^College of Medicine, The Ohio State University, Columbus, OH, United States; ^2^Division of Nephrology, The Ohio State University Wexner Medical Center, Columbus, OH, United States; ^3^Division of Hematology, The Ohio State University Wexner Medical Center, Columbus, OH, United States; ^4^Department of Neurology, The Ohio State University Wexner Medical Center, Columbus, OH, United States; ^5^Department of Oncology Rehabilitation, The Ohio State University Wexner Medical Center, Columbus, OH, United States; ^6^Division of Cardiology, The Ohio State University Wexner Medical Center, Columbus, OH, United States; ^7^Division of Hematology/Oncology, OhioHealth, Columbus, OH, United States; ^8^Cardio-Oncology Center of Excellence, Washington University in St. Louis, St. Louis, MO, United States

**Keywords:** amyloidosis, heart failure, light chain, pharmacology, transthyretin

## Abstract

**Background:**

Beta-adrenergic antagonists or blockers (BB) are a cornerstone of cardiac therapy for multiple indications. However, BB are considered relatively contraindicated in amyloid cardiomyopathy due to poor tolerance. This intolerance is hypothesized to be due to concomitant neuropathy and significant restrictive cardiomyopathy. This study analyzes the incidence and characteristics of BB tolerance in patients with amyloid cardiomyopathy.

**Methods:**

Through a single-center retrospective chart review, patients with amyloid cardiomyopathy, confirmed by endomyocardial biopsy or technetium-99 pyrophosphate scan, were identified and clinical data was collected. Statistical methods included Chi-square test and two sample *t*-tests.

**Results:**

Of 135 cardiac amyloidosis patients, 27 patients (20.0%) had no BB use, 56 patients (41.5%) were current BB users, and 52 patients (38.5%) were prior BB users. The most frequent indications for BB use were heart failure, hypertension, coronary artery disease, and arrhythmia. The most common reason for stopping BB therapy was hypotension (62.8%) followed by fatigue, bradycardia, and orthostasis. Neurologic symptoms at the initial BB prescription or most recent evaluation were not significantly different between current and prior BB users. Their cardiovascular profiles were similar by ejection fraction, wall thickness, troponin I, and brain natriuretic peptide. There was no association for BB discontinuation based on amyloid subtype, sex, or race.

**Conclusion:**

The majority of patients with amyloid cardiomyopathy were prescribed BB, and over half of these patients still tolerated BB therapy. Current and prior BB users had similar profiles from a cardiovascular and neurologic perspective, with no association identified to predict BB discontinuation.

## Introduction

Amyloid cardiomyopathy is increasingly being recognized as an under-diagnosed cause of heart failure. Through extracellular deposition of amyloid fibrils, cardiac amyloidosis produces a non-ischemic, restrictive cardiomyopathy, which initially manifests as diastolic heart failure and may progress to systolic dysfunction in later stages. Recent screening studies have highlighted the need for higher clinical suspicion for amyloidosis in the setting of heart failure due to a higher prevalence of amyloid cardiomyopathy in patients with heart failure than previously thought (1–3). Studies showing higher prevalence have helped to drive the development of new treatment modalities for amyloid cardiomyopathy and, more generally, for various systemic amyloidosis causes (4–7). New amyloidosis treatments have imparted increased importance in effectively managing organ-specific amyloid manifestations—such as amyloid cardiomyopathy—in order to extend survival (8).

Guideline-recommended medical management of cardiac amyloidosis sequela—such as heart failure, conduction system disease, and arrhythmias—can be difficult. Due to systemic amyloid involvement in peripheral and autonomic nerves, neuropathy can limit tolerance of neurohormonal medications, such as beta-adrenergic antagonists or blockers (BBs) (9). In addition to their utility in preventing adrenergic receptor downregulation in systolic heart failure, BBs also help prevent cardiac arrhythmias and are used for atrial fibrillation rate control (10). Arrhythmia is a frequent complication of amyloid cardiomyopathy with a prevalence as high as 40% in this disease population—including a 25% prevalence of atrial fibrillation—and correlates to poorer hospital outcomes, increased hospital length-of-stay, and increased hospitalization cost (11).

Currently, BBs are considered to be relatively contraindicated in the management of cardiac amyloidosis (3, 9, 12). BBs face hemodynamic intolerance and bradycardia risk in a heart that is prone to conduction system disease and may be relying on compensatory tachycardia for adequate cardiac output (13–15). BB tolerance has been associated with improved all-cause mortality in transthyretin amyloidosis (ATTR) and light chain amyloidosis (AL) in some studies (16), but not in others (17).

Novel amyloid treatment can slow disease progression and enhance survival for cardiac amyloidosis patients (9). These developments highlight the importance of exploring patient tolerance to and utility of guideline directed medical therapy (GDMT) in cardiac amyloidosis. For BB therapy's effect on the long-term clinical trajectory of cardiac amyloid patients to be investigated, these medications must be hemodynamically tolerated in the short-term. Through a retrospective observational study, we define the incidence of BB tolerance and the characteristics that may be associated with BB tolerance in patients with amyloid cardiomyopathy seen at our institution between 2008 and 2020.

## Materials and Methods

### Ethical Approval

The Office of Responsible Research Practices determined this study (2020E0998) exempt from institutional review board (IRB) review. In addition, the Ohio State University HIPAA Privacy Board granted the project a full waiver of HIPAA authorization by expedited review, according to 45 CFR 164.512.

### Participants

Patients with suspected cardiac amyloidosis were identified based on ICD-9 or ICD-10 codes between 2008 and 2020 at The Ohio State University Wexner Medical Center (OSU). Inclusion criteria were diagnosis with wild type transthyretin amyloidosis (ATTRwt) (E85.82), hereditary or variant transthyretin amyloidosis (ATTRv) (E85.2), or light chain amyloidosis (AL) (E85.81) with known cardiac involvement (E85.4); or diagnosis of heart failure (ICD-9: 428.^*^ ; ICD-10:150.^*^) plus diagnosis of amyloidosis (ICD-9: 277.3^*^ ; ICD-10 E85.^*^). Exclusion criteria were clinically unconfirmed disease. Additionally, a single patient was excluded due to a diagnosis of secondary amyloidosis (AA). Endomyocardial biopsy-derived pathology specimens or technetium-99 pyrophosphate scans were used to confirm disease for AL and ATTR amyloidosis. To increase internal validity and decrease selection bias, all cardiac amyloidosis patients seen at OSU during the study timeframe were evaluated for inclusion and exclusion criteria.

Upon meeting study criteria, patients were stratified to current, prior, and no BB use groups for analysis. Patients were grouped in this manner to facilitate comparison of the current-use and prior-use categories. Patients currently on BB therapy must be reasonably tolerating therapy, whereas prior BB users required discontinuation.

### Variables

Demographic variables collected were age, sex, and race. Amyloid type, subtype, and diagnosis date were collected to characterize disease. BB use was characterized by BB type, initiation and discontinuation dates, indication, and reason for discontinuation. Cardiac profiles included laboratory data (troponin and brain natriuretic peptide) and imaging data (ejection fraction, stroke volume, and septal wall thickness *via* echocardiogram). Neurologic involvement was assessed by collecting reported neurologic symptoms.

Neurologic and cardiac data were obtained at two separate timepoints when available. Specific timing of the two data collection points was based on the category of BB use pattern. For current BB users, data was collected at or near initial BB prescription date and at the most recently available datapoint. For prior BB users, data was collected at or near initial BB prescription date and at or near BB discontinuation date. For non-BB users, data was collected at or near initial amyloid diagnosis and at the most recently available datapoint. These time points were chosen for their ability to represent clinical change over the course of BB use and/or disease course. When possible, data was obtained on the exact relevant date (i.e., vital signs obtained from clinician note for an appointment in which a BB was prescribed). This proved difficult with some variables. Specifically, imaging and lab data were frequently gathered from the available date in nearest proximity to the desired data collection date.

All patients prescribed a BB were included in the current or prior category regardless of length of therapy. Indication data was collected at the point of initial BB prescription, and not subsequent BB medication changes or additions. BB discontinuation data was collected only for prior BB users at the time of final BB discontinuation, and not for BB that were switched to other BB or only temporarily discontinued. Data collection was reviewed by two investigators for accuracy.

### Analysis

Continuous variables were reported as mean (standard deviation) and differences between groups were assessed *via* unpaired Student's *T*-test. Categorical variables were reported as percentage (number) and differences between groups were assessed *via* chi-square test. Statistical significance was considered with a *P*-value <0.05. Stata software was used for all statistical calculations.

## Results

**Figure 1:**
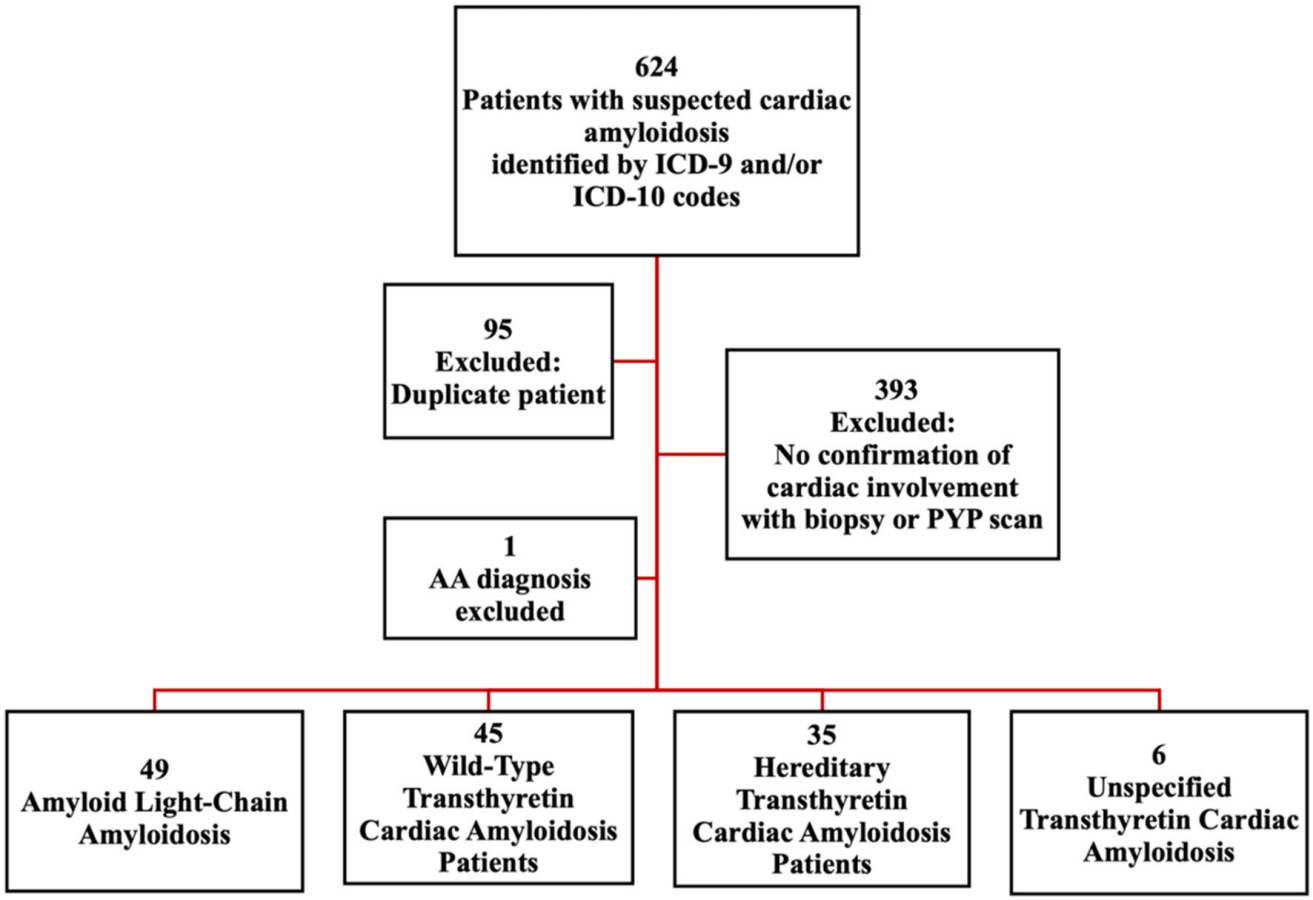
Schematic to identify cardiac amyloidosis patients. Identification of cardiac amyloidosis patients based on ICD codes: Criterion 1, Diagnosis with wild type Wild-Type Transthyretin Amyloidosis (wtATTR), Light Chain Amyloidosis (AL) or Hereditary Transthyretin Amyloidosis (hATTR), with known cardiac involvement. OR Criterion 2, Diagnosis of heart failure (ICD-9: 428.^*^ ; ICD-10: I50.^*^) plus diagnosis of amyloidosis (ICD-9: 277.3^*^ ; ICD-10 E85.^*^). AA, Secondary Amyloidosis; PYP scan, Technetium-99 Pyrophosphate Scintigraphy.

A total of 624 patients were identified to have suspected cardiac amyloidosis based on ICD-9 or ICD-10 codes between 2008 and 2020. Of these, 95 records were duplicate and 393 patients were excluded due to lack of confirmed cardiac involvement *via* endomyocardial biopsy (for either AL or ATTR amyloidosis) or technetium-99 pyrophosphate scan (for ATTR amyloidosis). Additionally, one secondary amyloidosis (AA) patient was excluded. We identified 135 patients with confirmed amyloid cardiomyopathy meeting inclusion and exclusion criteria. Of these, 49 had AL amyloidosis, 45 had ATTRwt amyloidosis, 35 had ATTRm amyloidosis, and 6 had unspecified ATTR amyloidosis (Figure 1).

Patients were stratified to current, prior, and no BB use groups for comparison and analysis (Table 1). In the current BB use category, there were 56 participants with a mean age of 72 years (SD = 11.28), 23.2% female, and 62.5% were Caucasian. In the prior BB use category, there were 52 patients with a mean age of 72 years (SD = 10.02), 26.9% female, and 67.3% were Caucasian. In the no BB use category, there were 27 patients with a mean age of 71 years (SD =11.23), 25.9% female and 85.2% Caucasian.

**Table 1:** Demographic data by beta blocker use pattern.

	**Current beta-blocker use**	**Prior beta-blocker use**	**No beta-blocker use**
Patients (n)	56	52	27
Mean age	71.80 ± 11.28	72.17 ± 10.02	70.73 ± 11.23
(years ± SD)
Age range (years)	42–96	48–93	44–91
Female (%)	23.20	26.90	25.90
Caucasian (%)	62.50	67.31	85.20

*Standard deviation (SD)*.

**Figure 2:**
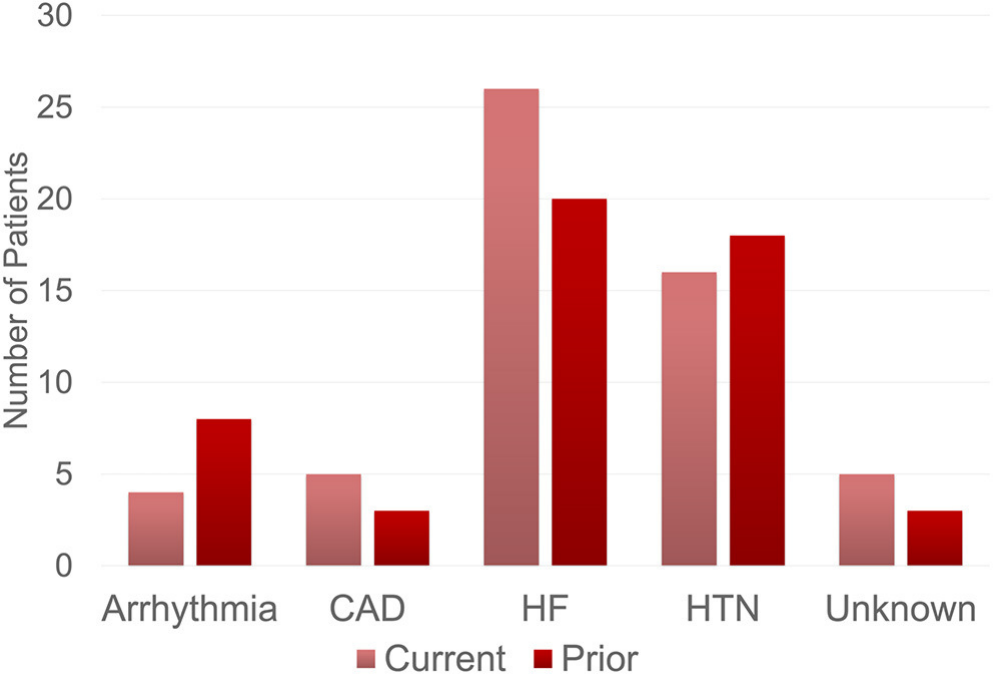
Beta-blocker indications. Indication for beta-blocker use among patients receiving current and prior beta-blocker therapy. The current group includes cardiac amyloid patients who were on beta-blocker therapy at time of data collection. The prior group includes cardiac amyloid patients who were previously on beta-blocker therapy but were no longer using beta-blockers at the time of data collection. The most common reason for beta-blocker therapy in both groups included heart failure followed by hypertension. CAD, Coronary Artery Disease, HF, Heart Failure; HTN, Hypertension.

**Figure 3:**
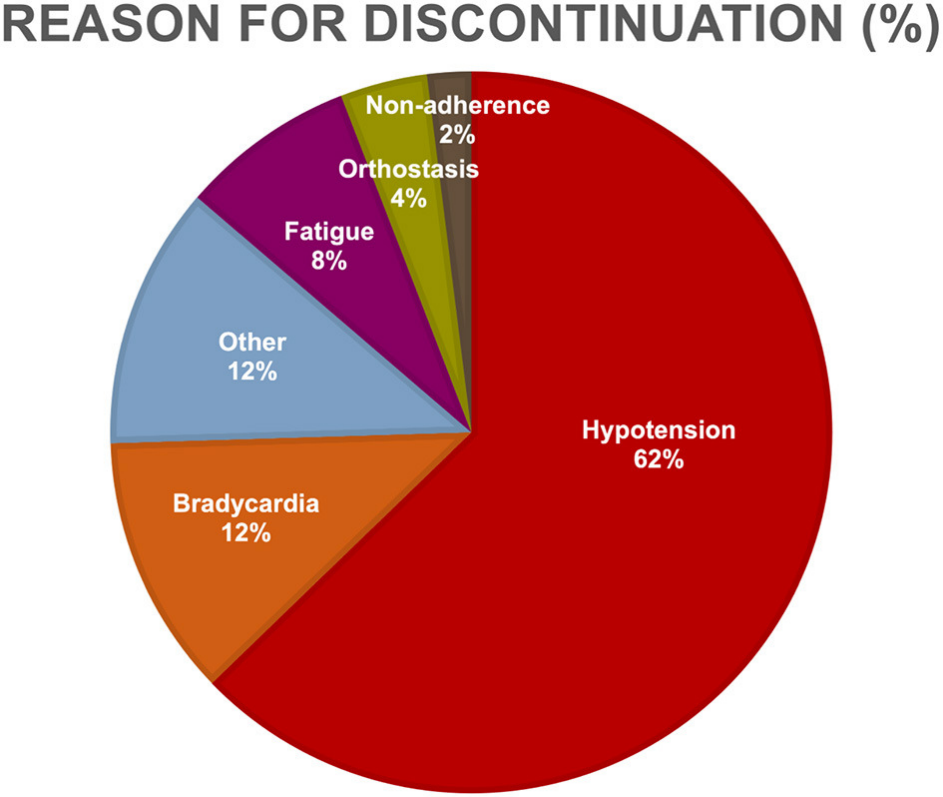
Reasons for beta-blocker discontinuation. Listed are the reasons for discontinuation of behhta-blocker therapy among cardiac amyloid patients. The most common reasons for stopping beta-blocker therapy were hypotension, bradycardia, fatigue, and orthostasis.

The most frequent BB indications were heart failure (46.4% vs. 38.5%), hypertension (28.6% vs. 34.6%), coronary artery disease (10.6% vs. 4.8%), and arrhythmias (8.9% vs. 5.8%), for current and prior BB use, respectively (Figure 2). For current and prior BB use group, there was no statistical difference in indication for BB initiation (χ^2^ = 3.09, *p*-value = 0.54), the proportion of patients initially placed on BBs before vs. after amyloid diagnosis, and the BB type (Table 2). The most common reasons for stopping BB therapy were hypotension (62.8%), bradycardia (11.8%), fatigue (7.8%), and orthostasis (3.9%) (Figure 3).

**Table 2:** Beta-blocker (BB) prescription type and timing in relationship to amyloidosis diagnosis.

	**Current BB use % (*N*)**	**Prior BB use % (*N*)**	**Test statistic**	***P*-value**
BB type^a^
Atenolol	8.9 (5)	7.7 (4)	*X*^2^ = 0.0539	0.816
Carvedilol	46.4 (26)	42.3 (22)	*X*^2^ = 0.1854	0.667
Metoprolol	69.6 (39)	80.8 (42)	*X*^2^ = 1.7802	0.182
Timing of initial BB prescription
Prior to amyloid diagnosis	73.2 (41)	77 (40)	X^2^ = 0.1978	0.657
After amyloid diagnosis	26.8 (15)	23 (12)		

a *only BB used by at least 5 patients were included*.

Multiple parameters were assessed to determine whether demographic or disease parameters could account for discontinuation of BB therapy. No difference was found between current and prior BB use groups in regard to for amyloid subtype, sex, and race (Table 3). Current and prior BB users' cardiovascular profiles were similar by echocardiogram parameters including ejection fraction (45% vs. 50%) and wall thickness (1.50 vs. 1.47 cm) and by cardiac biomarkers including troponin I (0.175 vs. 0.244 ng/mL), and brain natriuretic peptide (552 vs. 593 pg/mL). The presence of neurological symptoms at initial BB prescription was not associated with BB tolerance (Table 4).

**Table 3:** Comparison of demographic variables compared between patients with current vs prior beta blocker categories.

**Variable**	**Current BB use % (*N*)**	**Prior BB use % (*N*)**	**Test statistic**	***P*-value**
Amyloid subtype AL ATTRv ATTRwt ATTR unspecified	(56) 30 (17) 30 (17) 38 (21) 2 (1)	(52) 34 (18) 25 (13) 31 (16) 10 (5)	*X*^2^ = 3.76	0.288
Sex Male Female	(56) 77 (43) 23 (13)	(52) 73 (38) 27 (14)	*X*^2^ = 0.20	0.657
Race Caucasian Black Other	(56) 62 (35) 38 (21) (0)	(52) 67 (35) 31 (16) 2 (1)	*X*^2^ = 1.53	0.465

*Beta-blocker (BB), Light chain amyloidosis (AL), Variant or hereditary transthyretin amyloidosis (ATTRv), wild-type transthyretin amyloidosis (ATTRwt)*.

**Table 4:** Cardiac and neurologic variables by current and prior beta-blocker (BB) use.

**Variable**	**Current BB use Mean ±SD (N)**	**Prior BB use Mean ±SD (N)**	**Test statistic**	***P*-value**
Ejection fraction (%)
*Initial on BB*	45.36 ± 13.74 (48)	50.10 ± 11.92 (46)	*t =* 1.78	0.078
Ejection (%)
*Most recent on BB*	43.10 ± 14.39 (41)	43.20 ± 13.65 (47)	*t =* 0.03	0.972
Stroke volume (cm/ml)
*Initial on BB*	39.40 ± 14.03 (30)	49.11 ± 25.95 (31)	*t =* 1.81	0.075
Stroke volume (cm/ml)
*Most recent on BB*	43.86 ± 21.73 (24)	40.25 ± 16.88 (43)	*t =* −0.76	0.451
Septal wall thickness (cm)
*Initial on BB*	1.50 ± 0.43 (38)	1.47 ± 0.42 (40)	*t =* −0.36	0.721
Septal wall thickness (cm)
*Most recent on BB*	1.56 ± 0.39 (33)	1.64 ± 0.60 (45)	*t =* 0.62	0.540
Troponin I (ng/mL)
Initial on BB	0.18 ± 0.18 (53)	0.24 ± 0.36 (42)	*t =* 1.22	0.227
Troponin I (ng/mL)
*Most recent on BB*	0.79 ± 3.22 (48)	0.44 ± 0.95 (49)	*t =* −0.73	0.468
Brain natriuretic peptide (pg/mL)
*Initial on BB*	551.79 ± 469.40 (48)	593.06 ± 755.30 (41)	*t =* 0.31	0.754
Brain natriuretic peptide (pg/mL)
*Most recent on BB*	864.27 ± 857.18 (48)	785.71 ± 632.39 (49)	*t =* −0.51	0.608
Neurological symptoms at initial BB prescription % (*N*)	52% (29)	52% (27)	*X*^2^ = 0.0002	0.989

## Discussion

In this study of 135 patients at a US tertiary referral center with confirmed amyloid cardiomyopathy, the majority of patients (80%) were prescribed BBs with 41.5% of study patients as current BB users. Our study reports a much higher baseline prescription rate of BB than previously described, which may reflect differences in US practice patterns as well as differences in subtypes of amyloidosis, including ATTRv. In a retrospective study of Italian patients with ATTR cardiac amyloidosis, only 57% of patients were prescribed BB and 33% of patients continued on BB therapy (18). A Spanish study of 128 ATTR cardiomyopathy patients found 50.8% on BB therapy with an only 25% discontinuation rate (16). A Greek study of 53 patients with AL amyloid cardiomyopathy on BB therapy found that 47% discontinued therapy. (19) Intentional prescription of BB was able to increase patients on BB therapy from 61 to 87% without an increase in adverse events in a recent Italian study (20). The prevalence of BB use at final data collection point in our study correlates with other real-world, non-trial analyses of BB use among amyloid cardiomyopathy patients (21).

Investigation into factors associated with BB tolerance among amyloid cardiomyopathy patients has been limited (22). In our study, heart failure and hypertension comprised the majority of documented reasons for BB initiation. An Italian study of 642 patients with cardiac amyloidosis found BB prescription was driven primarily by atrial fibrillation or ventricular arrhythmias (18). This striking difference in initiation reasons may reflect higher prevalence of underlying hypertension in the US population. Left ventricular ejection fraction was also higher overall in the Italian study. Consistent with other clinical observations, hypotension was the most common reason for BB discontinuation in this study.

Between patients who were discontinued or continued on BB therapy, no significant differences in cardiac profiles, neurologic symptom incidence, amyloid type (AL or ATTR), BB type, or demographic data were found in our study. One study did find increased BB intolerance in patients with more advanced AL disease with higher NYHA class and Mayo stage (19). Nevertheless, few analyses have found significant association in BB intolerance—in part because the numbers are small.

Although our study did not find any significant differences between groups tolerant and intolerant to BB therapy, it does build on a prevalent tolerance of BB therapy in amyloid cardiomyopathy seen in the above studies. The number of patients both prescribed and tolerating BB therapy in our study demonstrates the clinical complexity formed by competing considerations of GDMT heart failure strategies and the specific conduction system and neurohormonal concerns in the amyloid population. Determining whether the long-term efficacy of BB in amyloid cardiomyopathy is equivalent to the GDMT benefit in other forms of heart failure remains unknown and is an imperative future study.

Limitations to this study include the retrospective nature at a single institution, which introduces the potential for measurement bias in relation to clinical care data. Though appropriate steps were taken to limit this, some data points were either missing or unable to be collected at the exact appropriate time. Missing laboratory data prevented reporting of clinical staging data, which may have proved useful to investigate relationships between disease severity and BB tolerance. Further, the impact of BB on outcomes could not be appropriately judged in this analysis. Although our study is relatively large in this underrecognized disease, our study lacks appropriate numbers to power subgroup analyses and investigate more specific types of amyloid patients who may best tolerate BBs.

**Figure 4:**
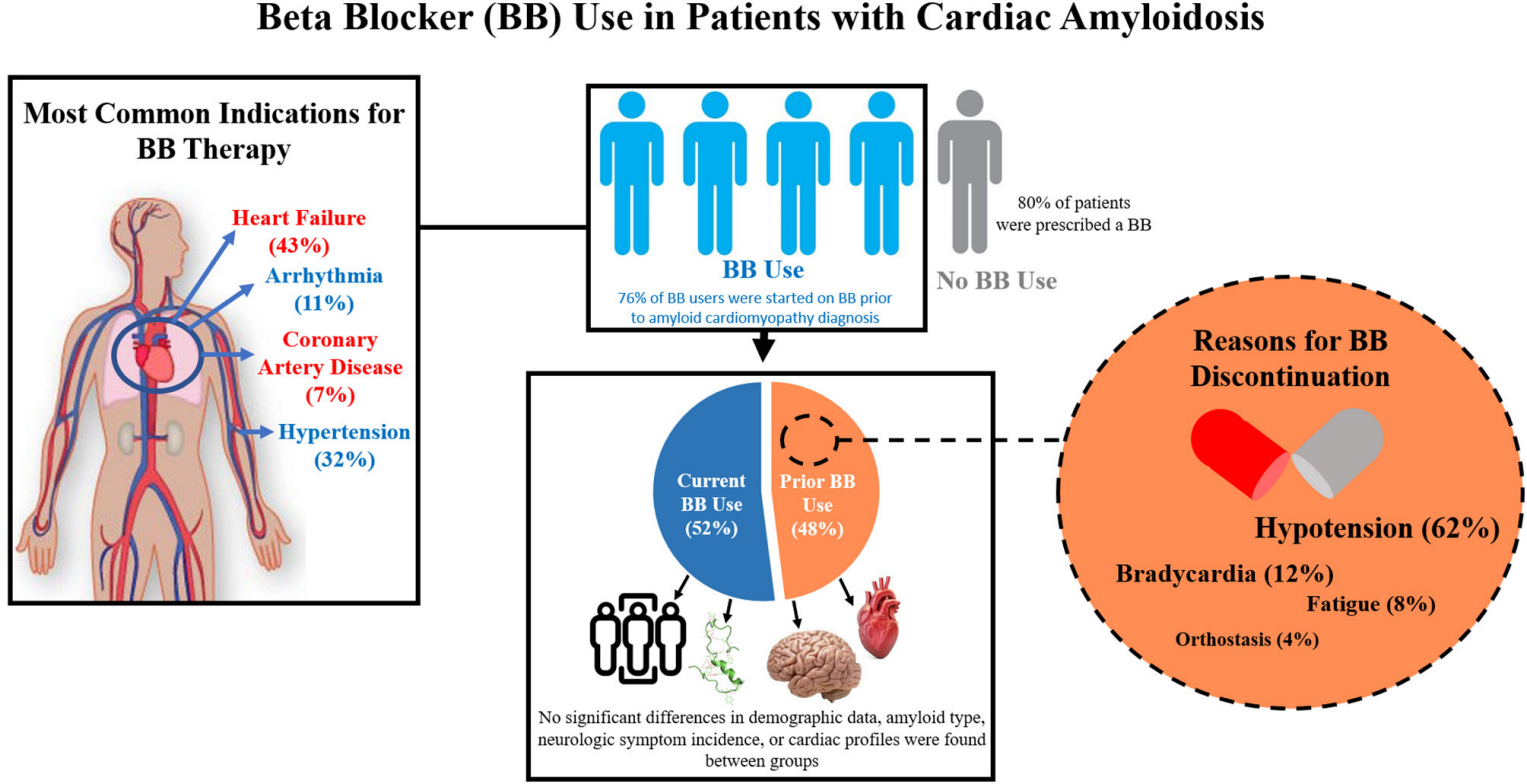
Summary Figure. Beta-blocker use and tolerance were analyzed in a cohort of patients with confirmed amyloid cardiomyopathy in a large amyloidosis referral center in the United States. The most common indications for beta blocker (BB) therapy in our cohort included heart failure, hypertension, arrhythmia, and coronary artery disease. Most patients in our study cohort were prescribed a beta blocker (BB). Of these, over half of them were tolerating the therapy enough to remain on the medication. Between patient groups tolerating (Current BB Use) and not tolerating (Prior BB Use) BB therapy, no significant differences in cardiac profiles, neurologic symptom incidence, amyloid type, or demographic data exist. The most common reasons for BB discontinuation include hypotension, bradycardia, fatigue, and orthostasis.

This study shows that in a cohort of 135 amyloid cardiomyopathy patients receiving care at an amyloid referral center in the US, the majority of patients were prescribed a BB (Figure 4). Furthermore, over half of patients prescribed a BB were tolerating the therapy enough to remain on the medication. Between patient groups tolerating and not tolerating BB therapy, no significant differences in cardiac profiles, neurologic symptom incidence, amyloid type, or demographic data exist. The most common reason for BB discontinuation was hypotension. In the context of amyloid cardiomyopathy, further study is needed to better understand which characteristics may be predictive of BB tolerance, ideal BB regimens for patients tolerating therapy, and the effects of BB therapy on cardiac disease progression.

## Data Availability Statement

The raw data supporting the conclusions of this article will be made available by the authors, without undue reservation.

## Ethics Statement

The studies involving human participants were reviewed and approved by the Office of Responsible Research Practices. Written informed consent for participation was not required for this study in accordance with the national legislation and the institutional requirements.

## Author Contributions

CC and RK conceived this study. SR, CA, BR, CT, and CC designed, analyzed, and interpreted the data. SR and CA wrote the first draft of the manuscript. All authors have contributed significantly to this work, manuscript revision, read, and approved the submitted version.

## Funding

This publication was supported, in part, by the National Center for Advancing Translational Sciences of the National Institutes of Health under Grant Numbers TL1TR002735 and UL1TR001450. CC was also supported by the Amyloidosis Foundation, Cardiac Amyloidosis Fellowship.

## Author Disclaimer

The content is solely the responsibility of the authors and does not necessarily represent the official views of the National Institutes of Health.

## Conflict of Interest

CC reports research support from Alnylam Pharmaceuticals, Akari Therapeutics, and Pfizer, Inc and consultant fees from Alnylam. The remaining authors declare that the research was conducted in the absence of any commercial or financial relationships that could be construed as a potential conflict of interest.

## Publisher's Note

All claims expressed in this article are solely those of the authors and do not necessarily represent those of their affiliated organizations, or those of the publisher, the editors and the reviewers. Any product that may be evaluated in this article, or claim that may be made by its manufacturer, is not guaranteed or endorsed by the publisher.
